# Multi-Copper Oxidases and Human Iron Metabolism

**DOI:** 10.3390/nu5072289

**Published:** 2013-06-27

**Authors:** Ganna Vashchenko, Ross T. A. MacGillivray

**Affiliations:** 1 Department of Cellular and Physiological Sciences, University of British Columbia, 2350 Health Sciences Mall, Vancouver, BC, V6T1Z3, Canada; E-Mail: ganna.vashchenko@gmail.com; 2 Department of Biochemistry and Molecular Biology, Centre for Blood Research, University of British Columbia, 2350 Health Sciences Mall, Vancouver, BC, V6T1Z3, Canada

**Keywords:** multi-copper oxidase, ferroxidase, ceruloplasmin, hephaestin, zyklopen

## Abstract

Multi-copper oxidases (MCOs) are a small group of enzymes that oxidize their substrate with the concomitant reduction of dioxygen to two water molecules. Generally, multi-copper oxidases are promiscuous with regards to their reducing substrates and are capable of performing various functions in different species. To date, three multi-copper oxidases have been detected in humans—ceruloplasmin, hephaestin and zyklopen. Each of these enzymes has a high specificity towards iron with the resulting ferroxidase activity being associated with ferroportin, the only known iron exporter protein in humans. Ferroportin exports iron as Fe^2+^, but transferrin, the major iron transporter protein of blood, can bind only Fe^3+^ effectively. Iron oxidation in enterocytes is mediated mainly by hephaestin thus allowing dietary iron to enter the bloodstream. Zyklopen is involved in iron efflux from placental trophoblasts during iron transfer from mother to fetus. Release of iron from the liver relies on ferroportin and the ferroxidase activity of ceruloplasmin which is found in blood in a soluble form. Ceruloplasmin, hephaestin and zyklopen show distinctive expression patterns and have unique mechanisms for regulating their expression. These features of human multi-copper ferroxidases can serve as a basis for the precise control of iron efflux in different tissues. In this manuscript, we review the biochemical and biological properties of the three human MCOs and discuss their potential roles in human iron homeostasis.

## 1. Introduction

### 1.1. Iron in Biology

Iron is an essential element in most biological systems. Only members of the *Lactobacillus* and *Bacillus* families can sustain life without iron [1]. The ability of iron to redox-cycle between its Fe(II) and Fe(III) forms is widely utilized in many biological processes. As a functional component of heme, iron participates in oxygen transport by hemoglobin [2] and drug detoxification by cytochrome P450 in liver [3]. When incorporated into iron-sulfur cluster proteins, iron can mediate mitochondrial electron transfer with the subsequent production of adenosine 5′ triphosphate (ATP) [4]. As a part of a binuclear site in ribonucleotide reductase, iron serves as an important factor in the synthesis of DNA [5]. In addition to these functions as a protein cofactor, iron has also been implicated as playing a role in the immune response [6].

Unfortunately, iron redox activity can also contribute to the production of hydroxyl radicals (via the Fenton series of reactions) and superoxide radicals [7]:

Fe^2+^ + O_2_ → Fe^3+^ + O_2_^−^ (1)

2O_2_^−^ + 2H^+^ → O_2_ + H_2_O_2_ (2)

Fe^2+^ + H_2_O_2_ → Fe^3+^ + OH^−^ + OH^•^ (3)

The hydrogen peroxide used in the Fenton reaction (3) is produced by superoxide dismutase and converts two superoxide molecules into oxygen and H_2_O_2_ (2), which then reacts with ferrous iron as above. Because the hydroxyl radical (OH^•^) and superoxide radical (O_2_^−^) have an unpaired electron on the outer orbital, they can be assigned to the group of reactive oxygen species (ROS). ROS can attack lipids, proteins and DNA, sometimes leading to cancer or cell death [8].

In addition to the high toxicity of Fe(II), the low solubility of Fe(III) at physiological pH is another obstacle for incorporation of iron in biological systems. At neutral pH and physiological oxygen tension, Fe(II) is readily oxidized into Fe(III). Under these conditions, Fe(III) tends to hydrolyze and forms the extremely insoluble Fe(OH)_3_ complex. Due to the low accessibility of this highly abundant metal, 66%–88% of the human population is affected by iron deficiency (World Health Organization Statistics, 2003, [9]).

Since both iron overload and iron deficiency cause cell death, the levels of biologically available iron must be tightly controlled. This set of conditions has led to the development of elaborate mechanisms of iron acquisition, trafficking and storage. 

### 1.2. Iron Metabolism in Humans

#### 1.2.1. Iron Absorption in the Small Intestine

**Figure 1:**
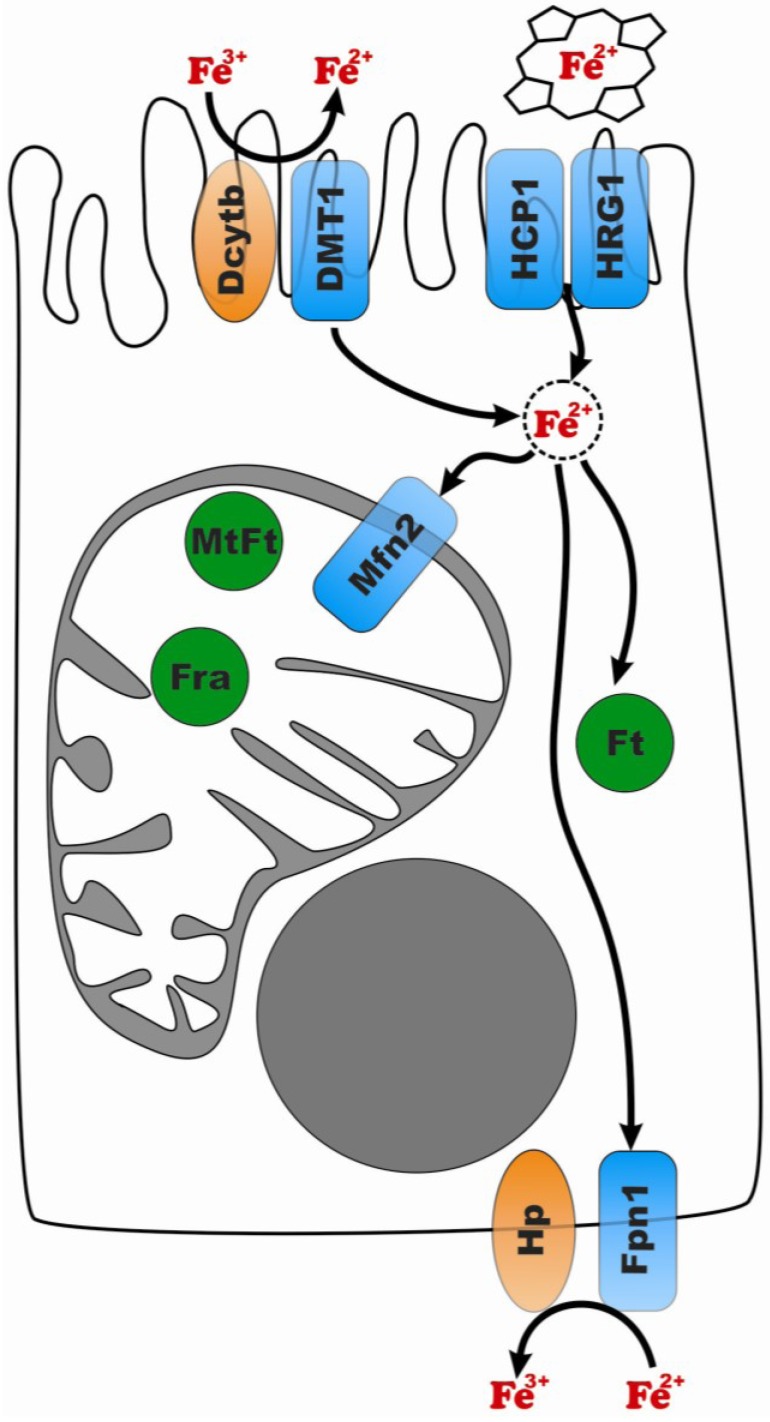
Transport of iron in the enterocyte. Proteins are shown with abbreviated names. Transporters are shows in blue; ferriductases and ferroxidases are depicted in orange; iron storage proteins are shown in green. Fe^2+^ in the dashed circle represents labile cytosolic iron pool.

Absorption of iron occurs in the proximal small intestine and is mediated by specialized epithelial cells called duodenal enterocytes (Figure 1). Iron can be absorbed from the diet as inorganic iron (iron salts or chelates) or as a part of heme, which is usually released after digestion of hemoglobin and myoglobin in dietary meat. Recent studies have also suggested a significant role of plant ferritins as an iron source in humans [10].

Two candidate heme transporters have been identified in the small intestine: heme carrier protein 1 (HCP1) [11] and heme responsive gene-1 (HRG-1) [12]. HCP1 is a member of a large family of proton-coupled transporters known as the major facilitator superfamily. Expression of both HCP1 and HRG-1 was detected in the small intestine, while HRG-1 is also expressed in the brain, heart and kidney [11,12]. It is not clear yet which of these transporters is predominant in dietary heme uptake. Regardless of the permease used for heme to cross the apical membrane of enterocytes, heme must be degraded for the iron to become metabolically available within the cell. Heme degradation is catalyzed by heme oxygenases and results in release of iron. Interestingly, induction of heme oxygenase 1 also causes an increase in HCP1 expression, suggesting a connection between uptake and degradation of heme [13].

Transport of inorganic iron is mediated by the divalent metal transporter DMT1 (also known as Nramp2, DCT1 and SLC11A2). DMT1 is a H^+^/divalent metal symporter that also transports other divalent metals (Zn^2+^, Cd^2+^, Mn^2+^, Cu^2+^, Co^2+^, Ni^2+^ and Pb^2+^) [14]. In addition to its function as a duodenal iron transporter, DMT1 is also responsible for iron release from endosomes in other cell types (see Section 1.2.2). Recent studies of SLC11A2 knockout mice have shown that DMT1 plays a significant role in intestinal iron absorption and iron uptake by erythroid cells, but the transporter is dispensable in placenta and liver [15]. Thus, DMT1 may be the primary means for iron transport, but it is not the sole mechanism.

To make iron available for transport by DMT1, Fe^3+^ must be reduced to Fe^2+^. The ferriductase activity on the apical surface of enterocytes has been attributed to duodenal cytochrome *b* (Dcytb) [16]. Dcytb is a di-heme protein, which is likely to use ascorbate as an electron donor [17]. In addition to this ferriductase function, Dcytb also has cupric reductase activity, providing a link between iron and copper metabolism [18,19]. Expression of this ferriductase is increased under conditions of iron deficiency, which serves as strong evidence of a role for Dcytb in iron uptake [16]. On the other hand, loss of this protein in Dcytb^−/−^ mice had little or no effect on body iron stores [20], which may imply involvement of some other ferriductase in duodenal iron absorption. 

When iron enters the enterocyte, it can be either stored or utilized for local needs or exported into the blood and delivered to the tissues. The main form of iron storage in humans is a ferritin encapsulated ferric hydroxide mineral. Structurally, ferritin resembles a cage composed of 24 subunits and is capable of storing up to 5000 iron atoms [21]. There are two types of ferritin subunits: H and L. Only the H subunit possesses ferroxidase activity and catalyses the rate-limiting step of iron incorporation into ferritin [21]. The L-subunit assists the ferroxidase activity of the H-subunit by promoting iron nucleation within the ferritin cavity [21]. The ratio between H and L subunits may vary depending on the cell type and physiological conditions [22,23].

Mitochondria represent another iron-enriched compartment in the cell. Both heme and Fe-S clusters are synthesized in mitochondria; this process requires high amounts of iron as well as the means for its safe handling. To date, two mitochondria-specific iron transporters have been reported: mitoferrin, required for efficient heme biosynthesis in erythroid cells [24], and mitoferrin 2, expressed in non-erythroid cells [25]. Inside the mitochondrion, iron can be captured by frataxin or mitochondrial ferritin. Due to its ability to bind iron, frataxin can function as an iron-storage protein or an iron chaperone during the production of heme and iron-sulphur clusters [26]. Mitochondrial ferritin (MtF) has properties similar to the H-subunit of cytosolic ferritin. While cytosolic ferritins are ubiquitous, expression of mitochondrial ferritin is mainly restricted to the testis, neuronal cells and islets of Langerhans [27]. The fact that these tissues are highly sensitive to ROS suggests a role of MtF in protecting mitochondria from iron toxicity.

For iron to exit the enterocyte, it has to be transported by the basolateral permease ferroportin 1 (Fpn1, also known as Ireg, MTP and SLC40A1). Ferroportin is the only known iron exporter in humans. In contrast to the iron uptake systems, which are ubiquitous throughout the body, only certain cell types have an iron export system. These cells play a major role in iron homeostasis (*i.e.*, duodenal enterocytes, macrophages, placental trophoblasts, hepatocytes and erythroblasts along with cells highly sensitive to ROS (neurons, β-cells in pancreas)). The observed embryonic lethality of Fpn1 null mice indicated that ferroportin is essential early in development [28]. Selective inactivation of Fpn1 in the small intestine, liver and macrophages caused iron accumulation in these tissues, confirming a unique role of Fpn1 as an iron exporter [28].

Fpn1 exports iron as Fe^2+^, but transferrin, the major iron transporter protein of blood, can bind only Fe^3+^ efficiently. This creates a need for a ferroxidase activity at the site of iron export. In enterocytes, this ferroxidase activity is associated with hephaestin, a putative multi-copper oxidase. The hephaestin ectodomain is highly similar to ceruloplasmin, a major ferroxidase of blood. In contrast to ceruloplasmin, hephaestin has a predicted transmembrane domain, which anchors this ferroxidase to the basolateral surface of enterocyte. Interestingly, GPI-linked ceruloplasmin is co-localized with Fpn1 on the surface of glial cells, producing an export system similar to the Fpn1-Hp system in the small intestine [29]. De Domenico *et al.* [30] have shown that ferroxidase activity stabilizes ferroportin in glial cells by preventing Fpn1 ubiquitination and its subsequent degradation. In the absence of ferroxidase activity, Fpn1 remains bound with Fe^2+^ which makes it accessible for ubiquitination. Oxidation of Fe^2+^ or use of Fe^2+^-specific chelators can abolish this effect [31]. It is worth noting that this mechanism of regulating the level of Fpn1 in the cell membrane is independent of Fpn1 degradation induced by hepcidin, an iron-regulatory peptide produced in liver [30] (for details see Section 1.2.3).

Taking into account the importance of immediate iron oxidation during iron export by ferroportin, it was anticipated that Fpn1 and membrane-anchored ferroxidases physically interact. Indeed, immunocytochemical analysis and immunoprecipitation experiments confirmed an interaction between ferroportin and GPI-linked ceruloplasmin in astrocytes [29] and a Fpn1-Hp interaction in enterocytes [32,33].

#### 1.2.2. Iron Uptake in Different Cell Types

Most of the iron in blood plasma is bound by transferrin, a glycoprotein with extremely high affinity for Fe^3+^ (K_D_ = 10^−21^ M) [34]. Under normal conditions, the concentration of transferrin iron-binding sites is greater than the concentration of iron, thereby ensuring a negligible amount of damaging free iron in the blood. As a means to arrange the direct and secure delivery of oxidized iron, an intimate interaction between the ferroxidase and the iron transport protein has been suggested. Detection of a 1:2 complex between ceruloplasmin and lactoferrin (the transferrin paralog in milk) provided the first evidence for this hypothesis [35,36]. Recent fluorescence emission spectroscopy experiments also confirmed a 1:1 complex formation between ceruloplasmin and transferrin [35], although surface plasmon resonance measurements did not detect any stable interaction between transferrin and recombinant hephaestin [37]. 

Iron binding to transferrin is pH-dependent, which allows efficient iron binding at the neutral pH of plasma but the intracellular release of iron at the low pH of the endosome (where transferrin is located after internalization [38]). Transferrin endocytosis is mostly mediated by transferrin receptor 1 (TfR1), a ubiquitously expressed membrane protein that binds holotransferrin with an affinity of 10^9^/M [39]. Another transferrin binding protein, TfR2, is restricted to hepatocytes, duodenal crypt cells and erythroid cells. TfR2 binds transferrin with an affinity 30-fold lower than TfR1 and may play a separate role in the regulation of iron homeostasis [40]. The acidic pH of endosomes stimulates iron release from transferrin with subsequent export of iron into the cytosol by DMT1. Because DMT1 transports only divalent cations and iron released from transferrin is in the Fe(III) form, the existence of an endosomal ferriductase was suggested. In erythroid cells, the main consumers of iron in the human body, this ferriductase function is performed by the protein Steap3 [41,42]. Ferriductases participating in iron release from endosomes in other cell types have not been reported yet.

Although Tf-dependent iron uptake is probably predominant under normal circumstances, in the case of iron overload (e.g., hereditary hemochromatosis and β-thalassemia), the iron binding capacity of transferrin can be exceeded. This situation results in the appearance of non-Tf-bound iron (NTBI). Previously known as a zinc transporter, the protein Zip14 was recently found to function as a transporter of NTBI in liver [43].

Megaline and cubilin are multi-ligand receptors which are primarily expressed in polarized epithelial cells. These proteins are co-expressed in the small intestine, renal proximal tubule and placental cytotrophoblast [44]. Because cubilin does not have any signals for endocytosis, it was proposed that megalin mediates co-internalization of cubilin. Cubilin binds transferrin, while both megalin and cubilin can bind hemoglobin. In the kidney, these binding interactions may be important for minimizing iron losses through the urine. Recently, megaline was suggested to have a new function related to iron homeostasis–binding of lipocalin (also termed neutrophil gelatinase-associated lipocalin, NGAL) [45]. NGAL is capable of binding certain types of bacterial siderophores [46]. By limiting the iron availability for pathogenic bacteria, NGAL works as a bacteriostatic agent [46]. Devereddy *et al.* [47] also suggested 24p3R as another candidate for the role of a lipocalin receptor.

Macrophages play an important role in iron homeostasis by recycling significant amounts of iron through the phagocytosis of old and damaged red blood cells [48]. Furthermore, haptoglobin and hemopexin (blood proteins which show high affinity for hemoglobin and heme, respectively) are endocytosed by macrophages through specialized receptors [49]. Iron recovered after heme degradation inside the macrophage is either held in storage or exported to reload circulating transferrin.

Despite the variety of iron uptake systems described above, biochemical data suggest that additional mechanisms for cellular iron uptake may exist. These mechanisms include iron uptake facilitated by putative ferritin receptors [50,51,52] or ceruloplasmin [53].

#### 1.2.3. Regulation of Iron Homeostasis

In humans, iron metabolism is regulated at both the cellular and systemic levels. At the cellular level, expression of proteins involved in iron homeostasis is modulated by affecting transcription, mRNA stability, translation and post-translational modifications [54]. Of these processes, post-transcriptional regulation is the best characterized. Iron regulatory proteins 1 and 2 (IRP1 and IRP2) are mammalian proteins that bind to iron-binding elements (IRE) in mRNA under iron deplete conditions. IREs in the 5′ untranslated region were identified in mRNAs encoding ferritin chains, erythroid 5-aminolevulinic acid synthase (the first enzyme of heme biosynthesis), mitochondrial aconitase (a citrate cycle enzyme) and one of the ferroportin isoforms [55,56,57]. Formation of an IRE/IRP complex in the 5′ UTR inhibits the early steps of translation. On the other hand, binding of IRP at the 3′ UTR of TfR1 mRNA and one isoform of DMT1 stabilizes RNA and enhances translation [58]. The intracellular iron concentration affects the binding of IRP1 and IRP2 through distinct mechanisms. IRP1 senses iron status through an iron-sulfur switch mechanism, alternating between an aconitase form with an iron-sulfur cluster assembled and an apoprotein form that binds IREs. IRP2 activity is regulated primarily by iron-dependent proteosomal degradation in iron-replete cells. Targeted deletions of IRP1 and IRP2 in animals demonstrated that IRP2 is the main physiologic iron sensor [59]. The central role for IRP-mediated regulation is supported by the early death of mouse embryos lacking both IRP1 and IRP2 [60].

In addition to this intracellular regulation, iron homeostasis is also coordinated at the organism level. Hepcidin, an iron-regulatory hormone expressed in the liver, is responsible for systemic regulation of iron homeostasis [61]. Upon binding to ferroportin, the sole iron exporter in humans, hepcidin induces its internalization and subsequent degradation [62]. Thus, by acting on ferroportin, hepcidin controls the three main entries of iron into plasma: (1) from duodenal enterocytes absorbing dietary iron, (2) from macrophages involved in the recycling of iron from erythrocytes, and (3) from hepatocytes involved in iron storage.

#### 1.2.4. Inherited Disorders of Human Iron Metabolism

Numerous mutations in the genes encoding proteins of iron metabolism have been reported (Table 1). The resulting dysfunctions of iron homeostasis lead to a variety of human disorders with mild to severe symptoms. While these mutations and associated phenotypes provide valuable insight into the mechanisms of iron homeostasis, they also emphasize the importance of studying iron metabolism for the development of new therapeutics.

**Table 1:** Hereditary disorders associated with iron imbalance.

Gene	Function of the protein	Disorder	Phenotype	References
DMT1	Ferrous iron transporter	Multiple missense mutations	Iron deficiency anaemia	[63,64,65]
H-ferritin	Iron storage	Mutation in 5′ UTR	Iron loading	[66]
L-ferritin	Iron storage	Neuroferritinopathia Hyperferritinaemia	Brain iron overload Cataract	[67,68,69]
Frataxin	Iron chaperone	Freidreich ataxia	Mitochondrial iron overloading	[70]
Ferroportin	Ferrous iron exporter	Hemochromatosis type 4	Plasma hypoferraemia with tissue iron loading	[71]
Ceruloplasmin	Systemic iron oxidase	Aceruloplasminaemia	Plasma hypoferraemia with tissue iron loading	[72]
Transferrin	Plasma iron transport protein	Atransferrinaemia	Anaemia refractory to iron therapy	[73,74]
TfR2	Uptake of transferrin Regulator of iron homeostasis	Hemochromatosis type 3	Iron loading	[75]
HFE	Regulator of iron homeostasis	Hemochromatosis type 1	Iron loading	[76]
Hemojuvelin	Regulator of iron homeostasis	Juvenile hemochromatosis (type 2A)	Iron loading	[77]
Hepcidin	Regulator of iron homeostasis	Juvenile hemochromatosis (type 2B)	Iron loading	[77]

### 1.3. Structure and Catalytic Mechanism of Multi-Copper Oxidases

Multi-copper oxidases (MCOs) are enzymes that oxidize their substrates with the concomitant reduction of dioxygen to two water molecules. Among other copper proteins, the unique feature of MCOs is the presence of at least one of each of the three types of copper sites: type 1, type 2 and binuclear type 3 [78]. This classification of protein copper sites is based on their spectroscopic and magnetic features that reflect the geometric and electronic structure of the copper-binding sites. A type 1 copper site shows intense absorption at around 600 nm and narrow hyperfine splitting in the electron paramagnetic resonance (EPR) spectrum. A type 2 copper site exhibits no absorbance maximum in the visible region of the spectrum but exhibits hyperfine splitting of normal magnitude in the EPR spectroscopy. Unlike type 1 and type 2 copper sites, a type 3 copper site is EPR-silent owing to the strong anti-ferromagnetic coupling. In the UV-visible spectrum, a type 3 copper site exhibits an absorbance maximum at 330 nm.

MCOs contain two, three or six cupredoxin domains, which consist of a mixture of antiparallel and parallel β-strands [79]. Three- and six-domain MCOs can function as a monomer while two-domain MCOs possess oxidase activity only when assembled as a homotrimer [80,81]. Most MCOs are composed of three domains with type 1 copper in domain 3 and a trinuclear cluster at the interface of domains 1 and 3. Type 1 copper serves as an acceptor of electrons from the substrate while the trinuclear cluster, comprising a type 2 and a binuclear type 3 centre, operates as a site of dioxygen reduction to water. Six-domain MCOs such as ceruloplasmin (and as predicted for hephaestin) contain type 1 copper atoms in domains 2, 4 and 6 and a trinuclear cluster at the interface of domains 1 and 6 [82].

Generally, MCOs are promiscuous with regard to their reducing substrate. Aromatic amines and phenols represent substrates of laccases [83] while ascorbic acid oxidase shows specificity towards ascorbic acid [84]. The small group of MCOs designated as metallo-oxidases exhibit an additional reactivity towards transition metals—Fe^2+^, Cu^+^, Mn^2+^ [85,86,87].

#### 1.3.1. Type 1 Copper Sites

Amino acid ligands normally found in the coordination sphere of type 1 copper sites of MCOs are two histidine and cysteine residues as equatorial ligands and a methionine residue as an axial ligand; the methionine residue may be substituted by non-coordinating leucine or phenylalanine residues [78]. Multiple studies have shown that the nature of the axial ligand in the type 1 copper center is a strong modulator of the copper reduction potential [88,89,90]. Copper coordination by a methionine residue results in a relatively low potential, while substitution of methionine with a non-coordinating residue leads to a significant increase in copper potential [90]. Second sphere ligands have also been suggested to affect the potential of type 1 copper in MCOs [91].

Coordination by protein ligands also affects the electronic structure of type 1 copper. Cu(II) harbors 9 5d-electrons with an unpaired d*_x_*_2−*y*2_ electron. Significant overlap between the d*_x_*_2−*y*2_ orbital of copper and the S_p_^−^ orbital of a coordinating cysteine allows for a charge-transfer, in which a large fraction of electronic charge of the electronic donor (Cys(S_p_^−^)) is transferred to the electron acceptor (the d*_x_*_2−*y*2_ orbital of type 1 copper). This charge-transfer results in a band of high intensity (ε~5000 M^−1^cm^−1^) that is visible in the absorption spectrum at 600 nm and is responsible for the intense blue color of MCOs [78,92].

#### 1.3.2. Transfer of Electrons to the Trinuclear Cluster and Dioxygen Reduction

Electron transfer from the type 1 copper (Cu1) center to the trinuclear cluster passes through the histidine-cysteine-histidine (H-C-H) triad, where a cysteine residue is a ligand of Cu1 and the histidine residues coordinate Cu3_a_ and Cu3_b_ (binuclear type 3 copper atoms). On its way from Cu1 to the trinuclear site, an electron passes through a distance of 13 Å using a through-bond mechanism [93].

**Figure 2:**
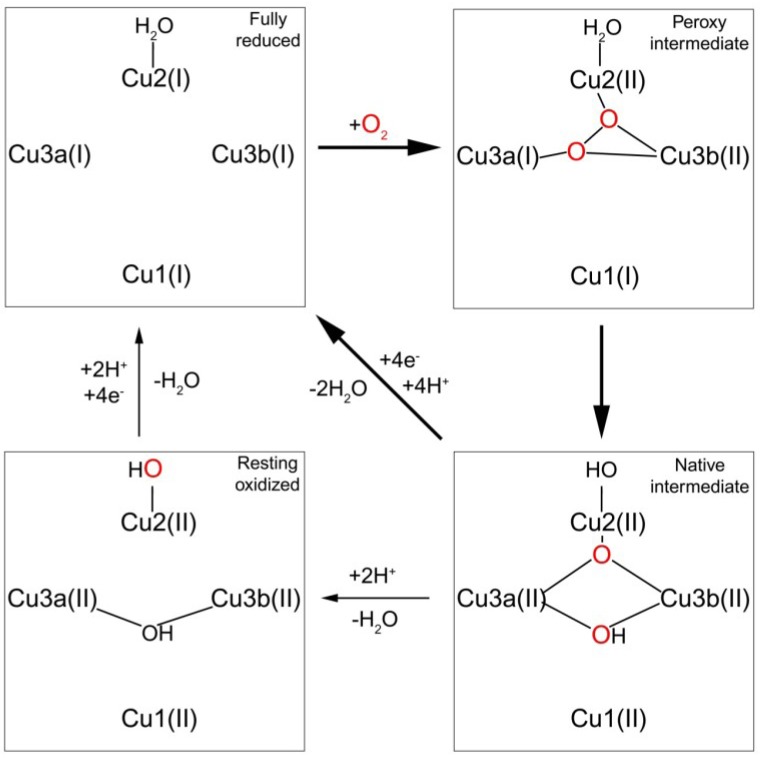
Mechanism of O_2_ reduction to water by the multi-copper oxidases (MCOs). Broad arrows indicate the steps that take place in the catalytic cycle of the MCO. Thin arrows indicate steps that can be experimentally observed but are not part of the catalytic cycle. Cu1 and Cu2 represent type 1 and type 2 copper, respectively, Cu3a and Cu3b are type 3 copper atoms. See [97] for a full discussion.

Copper atoms of the trinuclear cluster are arranged in a triangular fashion with six histidine residues coordinating the Cu3 pair and two histidine residues coordinating the type 2 copper (Cu2). Cu3_a_ and Cu3_b_ possess inequivalent second sphere ligands. The H-bond network created by a conserved aspartic acid residue lowers of the potentials of Cu2 and Cu3_b_ [94]. This effect allows the reduction of dioxygen in two sequential two-electron steps (Figure 2). First, the fully reduced MCO transfers two electrons to O_2_ to form a peroxy intermediate [95]. At this stage, the copper atoms with the higher potential (Cu1 and Cu3_a_) remain reduced. The remaining two electrons are then delivered to the peroxy intermediate to form the native intermediate. Decay of the native intermediate to H_2_O proceeds via successive proton assisted steps [96].

## 2. Human Multi-Copper Oxidases

To date, three MCOs have been identified in human body: ceruloplasmin (representing the MCO of blood), hephaestin (mainly expressed in small intestine), and zyklopen (the placental MCO). In addition to the oxidation of organic substrates, all human MCOs can oxidize ferrous iron (Fe(II)).

### 2.1. Ceruloplasmin

Ceruloplasmin was first purified from blood plasma by Holmberg and Laurell in 1948 [98]. The name “ceruloplasmin” literally means “a blue substance from plasma”. After discovery of the enzymatic activity of ceruloplasmin, some authors proposed (unsuccessfully) to change its name to “ferroxidase” [99]. Ceruloplasmin is an abundant glycoprotein in human plasma and is mainly produced by the liver [100]. In addition to its soluble form, GPI-anchored ceruloplasmin has been found in glial cells (CNS and retina) and Sertoli cells (testis) [101,102,103].

**Figure 3:**
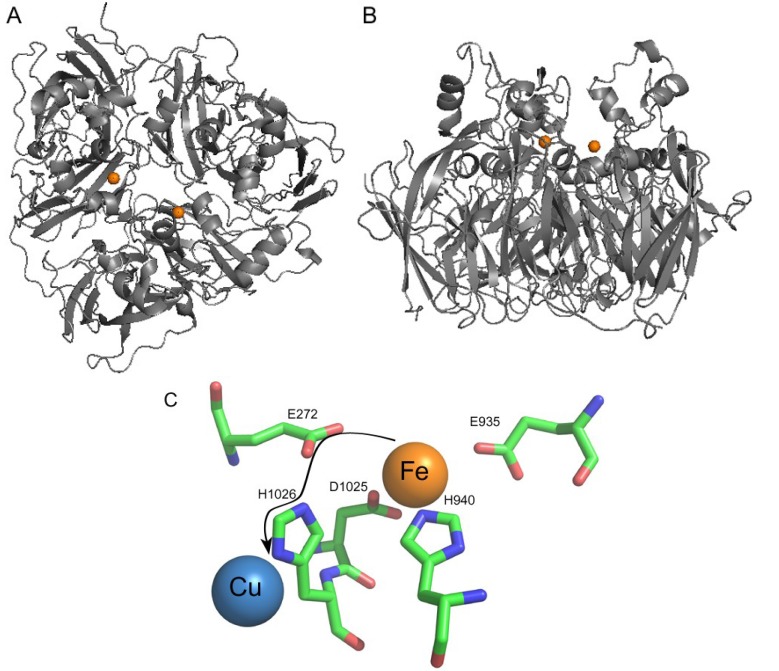
(**a**) The ribbon diagram of human ceruloplasmin. Top view of the molecule along the pseudo-3-fold axis. (**b**) Side view of ceruloplasmin almost perpendicular to the pseudo-3-fold axis with the putative iron binding site. (**c**) Iron-binding site in domain 6 of ceruloplasmin. Residues E272, E935, H940 and D1025 represent iron ligands, residue H1026 is a ligand of type 1 copper in domain 6. Arrow shows putative electron transfer path from the iron atom to the adjacent type 1 copper atom. Figures were generated using Pymol software (PDB ID 1KCW, Schrödinger, Portland, OR, USA).

**Figure 4:**
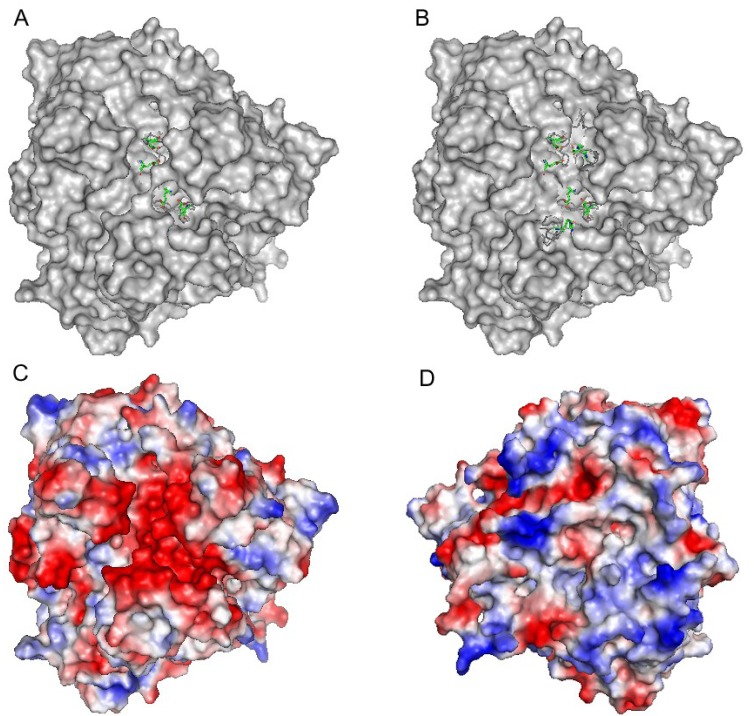
(**a**) Surface model of ceruloplasmin, top view of the molecule; predicted ligands of high-affinity iron-binding sites in domain 4 (E597, H602, D684, E971) and in domain 6 (E935, H940, D1025, E272) are shown as sticks. (**b**) Same as A; protein loops that cover iron-binding sites are shown as ribbons; groups of residues at the top and at the bottom represent iron-binding sites in domains 6 and 4 respectively. (**c**) Surface charge distribution on human ceruloplasmin. Top view of the molecule along the pseudo-3-fold axis. (**d**) Surface charge distribution on human ceruloplasmin. Bottom view of the molecule along the pseudo-3-fold axis. The negative and positive electrostatic potential regions are scaled from red for −76 to blue for +76 kT/e. The crystal structure of ceruloplasmin (PDB code 2J5W) was visualized with Pymol; the electrostatic map was obtained using APBS plug-in.

Ceruloplasmin contains six cupredoxin domains and has a molecular weight of 120 kDa. Type 1 copper centers are located in domains 2, 4 and 6 and a trinuclear cluster is formed between domains 1 and 6. The three-copper cluster is critical not only to the catalytic activity of ceruloplasmin, but also to the structural stability of the protein because it holds together the *N*- and *C*-terminal domains of holo-ceruloplasmin conferring a globular shape to this protein [104]. As revealed by crystal soaking experiments, ferrous binding sites are located in the vicinity of the type 1 copper atoms in domains 4 and 6 [105] (Figure 3). The putative iron ligands of ceruloplasmin are buried ~10 Å beneath the protein surface at the bottom of a narrow channel that limits access of bulky organic substrates. Due to the abundance of acidic amino acid residues, these predicted iron-binding sites and the surrounding protein surface possess significant negative charge (Figure 4c). Both putative iron-binding sites are composed of two glutamate, one aspartate and one histidine residue. As shown by near-infrared magnetic circular dichroism (near-IR-MCD), Fe^2+^ bound by ceruloplasmin is six-coordinated, suggesting the presence of two water molecules as additional iron ligands [106]. The iron-binding site in domain 6 of ceruloplasmin is comprised of E272, E935, H940 and D1025 with the last three residues contributed by domain 6 and the first one supplied by domain 2. Due to its hydrogen-bonding with H1026, which coordinates the type 1 copper in domain 6, E272 was predicted to participate in electron transfer between the iron-binding site and the adjacent type 1 copper site [106] (Figure 3c). In domain 2 of human ceruloplasmin, the residues that correspond to iron ligands in domains 4 and 6 are two glutamate residues, one aspartate residue and one tyrosine residue; these residues are not expected to form a ferrous binding site. In addition, the type 1 copper in domain 2 has a sufficiently high reduction potential that it cannot be oxidized without damaging the protein [107]. While involvement of domain 2 in the ferroxidase activity of ceruloplasmin remains unconfirmed, the functionality of iron-binding sites in domains 4 and 6 was recently supported by experimental data [108].

In addition to its role as a ferroxidase, ceruloplasmin exhibits several other catalytic activities. For example, ceruloplasmin was reported to have both NO-oxidase and glutathione-peroxidase activities [109,110]. As Cu^2+^ is regarded as the less toxic form of copper, ceruloplasmin cuprous oxidase activity has been suggested to play an important role in copper detoxification [111]. The prooxidant site of domain 2 of ceruloplasmin has been implicated in the oxidation of low-density lipoprotein (LDL) [112]. Furthermore, ceruloplasmin is capable of oxidizing an extensive group of organic substrates that includes both xenobiotics (organic amines) and physiologically relevant substrates (biogenic amines) [113,114]. The latter group includes hormones (adrenaline, noradrenaline) and neurotransmitters (serotonin, dopamine). Crystal soaking experiments revealed separate binding sites for these two groups of organic substrates. Organic substrates bind ceruloplasmin at domain 4 while the binding site for biogenic amines is located in domain 6 [115].

Although ceruloplasmin has been suggested to possess multiple physiological functions including roles in copper transport and oxidation of biogenic amines, studies involving aceruloplasminaemia patients revealed the major role of ceruloplasmin in iron metabolism. Aceruloplasminaemia is an autosomal recessive disease caused by mutations in the ceruloplasmin gene [116]. Most reported mutations result in premature termination of ceruloplasmin mRNA translation [117,118], while recently-found missense mutations affect ceruloplasmin trafficking and copper loading [119,120]. Overall, the critical physiologic defect in aceruloplasminaemia is the absence of enzymatically-active holoceruloplasmin. Confirming the role of ceruloplasmin in iron export, aceruloplasminaemic patients develop massive accumulation of iron in various tissues, including the liver, pancreas and brain [72,116]. Long-term iron accumulation leads to diabetes, retinal degeneration and neurologic symptoms in affected individuals [72,116]. These symptoms can be explained by iron toxicity, which results in free radical damage through the Fenton chemistry [121].

### 2.2. Hephaestin

Hephaestin was first discovered by Vulpe *et al.* [122] while studying the sex-linked anemia (*sla*) mouse. *Sla* mice develop microcytic hypochromic anemia with iron accumulation in the intestinal epithelium [123], suggesting that while apical iron intake is not impaired, iron export from enterocytes into the blood is blocked. By using positional cloning, the *sla* candidate gene was identified and named hephaestin after the Greek God of metalworking, Hephaestus [122].

Hephaestin is predicted to be a transmembrane protein with a molecular weight of approximately 130 kDa [124], and it was first detected in the small intestine [122,125]. Recent immuno-histochemistry experiments using an anti-Hp antibody have shown that Hp is also expressed in the antral portion of the stomach, the nerve plexi of the gastrointestinal tract and in human pancreatic β-cells [126]. The role of hephaestin in these other sites is unclear at present but may be associated with some sort of protection against damage by ROS (see later section).

The predicted amino acid sequence of human hephaestin is 50% identical and 68% similar to the sequence of human ceruloplasmin [124]. In contrast to its soluble serum homolog, hephaestin also contains a predicted transmembrane domain at the *C*-terminus. Based on the known crystal structure of ceruloplasmin, comparative structural modeling of the hephaestin ectodomain revealed that, with the exception of the axial type 1 copper ligand in domain 2, all residues involved in copper binding as well as all cysteinyl residues involved in disulfide bond formation in ceruloplasmin are conserved in hephaestin [124]. Unfortunately, coordinates for this hephaestin model [124] are not available from the protein data base.

In conjunction with iron transporter ferroportin, hephaestin mediates iron efflux from enterocytes and into the blood. By oxidizing ferrous ions, hephaestin promotes iron binding by transferrin and ensures efficient delivery of this metal to the tissues. The physiological importance of hephaestin-catalyzed ferroxidation is illustrated by the *sla* mice phenotype in which iron export from intestinal epithelium to the circulation is significantly impaired [123]. While hephaestin is mainly expressed in the intestine, this protein has recently been found in the placenta, heart, brain and pancreas [122,125,126,127,128]. In placenta hephaestin has been suggested to facilitate iron transfer between mother and fetus [129], whereas in heart, brain and pancreas ferroxidase activity of hephaestin can protect these tissues from Fe(II) toxicity.

Recent studies with recombinant hephaestin revealed new data on the catalytic mechanism and the substrate specificity of this protein. The *K*_m_ values of recombinant hephaestin for such organic substrates as *p*-phenylenediamine and *O*-dianisidine were close to values determined for ceruloplasmin [130]. However, in contrast to ceruloplasmin, hephaestin was incapable of direct oxidation of biogenic amines, such as adrenaline and dopamine [130], implying a difference in biological substrate specificities between these two homologous oxidases. In addition, kinetic studies revealed that similar to ceruloplasmin, hephaestin has two types of iron-binding sites with different affinities towards ferrous iron [131]. Studies involving site-directed mutagenesis confirmed that residues E960 and H965 serve as iron ligands of a high-affinity binding site located in domain 6 of hephaestin [131]. Based on homology with ceruloplasmin, the remaining ligands of this high-affinity iron-binding site are residues E300 and D996. Thus, the high-affinity iron-binding site in domain 6 of hephaestin is likely to be composed of a canonical set of ligands––three acidic residues and one histidine residue. 

The nature of the low-affinity iron-binding site(s) in both hephaestin and ceruloplasmin is less clear. At the top of the molecule, ceruloplasmin has a negatively charged patch that hosts two high-affinity binding sites [105] and may also accommodate the low-affinity binding site(s) (Figure 4c). Acidic residues of this negatively charged area are contributed by all six domains of ceruloplasmin. The high structural homology with ceruloplasmin along with similar kinetic behavior predicts similar structure of the low-affinity binding site(s) in hephaestin.

### 2.3. Zyklopen

Zyklopen is another human six-domain multi-copper ferroxidase [132]. This protein was detected in multiple tissues with the major site of expression being the placenta [132]. Physiological data implies that zyklopen is responsible for the iron efflux from placental cells [132,133,134]. Structurally, zyklopen is expected to be most closely related to hephaestin because both possess a putative transmembrane region at the *C*-terminus and have identical copper ligands [132,134]. In domain 6, zyklopen harbors a putative high-affinity iron-binding site that comprises amino acid residues that are highly conserved between human multi-copper ferroxidases. The same set of putative iron ligands occurs in domain 6 of hephaestin and ceruloplasmin. As a reflection of its similarity to hephaestin, zyklopen was named after Zyklops, the mythical one-eyed iron workers who helped Hephaestus in the forge of the gods. 

### 2.4. The Interplay between Human Multi-Copper Ferroxidases

Since the discovery of hephaestin and the more recent identification of zyklopen, ceruloplasmin is not considered to be the unique ferroxidase that facilitates iron export from the cells. The presence of several genes encoding proteins with ferroxidase activity emphasizes the importance of ferroxidation in iron metabolism but also raises the question about the particular function of each ferroxidase.

The distribution of MCOs in the human body ensures redundancy, with two ferroxidases present at most sites of expression, and all multi-copper ferroxidases are expressed in retina [102,132]. This observation suggests that compensatory relationships exist between the human ferroxidases. Indeed, in Cp^−/−^ mice, hephaestin compensates for the lack of ceruloplasmin ferroxidase function in defined regions of the brain [135]. This region-specific compensation may also explain iron accumulation associated with certain parts of the brain in aceruloplasminaemia patients [135]. Double knockout mice (Cp^−/−^Hp^sla/Y^) represent another useful tool for studying functional cooperation between these ferroxidases. While single knockout mice had a very mild iron loading phenotype [136,137], a cumulative effect was clearly observed in the double knockout mice. Cp^−/−^Hp^sla/Y^ mice developed severe iron overload in the pancreas, heart, brain and retina, suggesting the cooperation of ceruloplasmin and hephaestin in these tissues [72,102]. In contrast, these studies showed that iron efflux from the liver is facilitated solely by ceruloplasmin [72]. The age-dependent changes in phenotype of *sla* mice may provide another illustration of a compensatory link between ceruloplasmin and hephaestin. Young *sla* mice have severe anemia with symptoms decreasing with age [137]. Anemia of newborn mice may be explained by insufficient iron feeding of the fetus due to decreased ferroxidase activity of hephaestin in the placenta [129]. While ceruloplasmin is unable to compensate for hephaestin function in placental iron efflux [133,138], it can promote iron export from enterocytes [139]. This compensatory effect of ceruloplasmin in enterocytes can explain the weakening of anemic symptoms in adult *sla* mice.

Ceruloplasmin is the only ferroxidase that is expressed in a soluble form. Thus, as an abundant protein in blood, ceruloplasmin can perform many systemic functions. As a result of its important role in iron export and detoxification, ceruloplasmin transcription is up-regulated under conditions of iron deficiency and oxidative stress [140,141]. Due to the function of ceruloplasmin as an acute phase protein, its expression is also affected by cytokines such as interferon and interleukin 1β [142,143]. On the other hand, the expression and intracellular localization of hephaestin is regulated by iron in the intestine [144,145]; in contrast, the effect of iron on hephaestin levels in the heart was found to be negligible [127]. In the intestine, hephaestin expression is regulated by CDX2, a transcription factor with a key role in intestinal development and differentiation [146]. Expression of both hephaestin and zyklopen is modulated by copper [132,147,148], whereas plasma ceruloplasmin content remains unchanged in copper-deficient rats [149].

In conclusion, ceruloplasmin, hephaestin and zyklopen show distinctive expression patterns and have unique mechanisms for regulating their expression. These features of human multi-copper ferroxidases can serve as a basis for precise control of iron efflux in various tissues.

## 3. Conclusions

While being an indispensable element in many biological entities, iron can also be damaging due to the production of ROS through Fenton chemistry. A complex system for the safe handling of iron has evolved in the human body. As part of this system, three multi-copper ferroxidases protect cells and tissues from the harmful effects of ferrous iron by converting it into Fe(III). In addition to detoxification of iron, human MCOs also facilitate such important processes as iron absorption in the small intestine, transfer of iron from mother to fetus and iron release from liver and macrophages. The presence of multiple MCO paralogs confirms their important role in human iron metabolism and grants the additional plasticity in the regulation of iron toxicity and export from the cell.

## References

[1] Crichton R.R., Pierre J.L.  (2001). Old iron, young copper: From Mars to Venus. Biometals.

[2] Ponka P. (1999). Cell biology of heme. Am. J. Med. Sci..

[3] Danielson P.B. (2002). The cytochrome P450 superfamily: Biochemistry, evolution and drug metabolism in humans. Curr. Drug Metab..

[4] Lill R., Muhlenhoff U. (2006). Iron-sulfur protein biogenesis in eukaryotes: Components and mechanisms. Annu. Rev. Cell Dev. Biol..

[5] Eklund H., Uhlin U., Farnegardh M., Logan D.T., Nordlund P. (2001). Structure and function of the radical enzyme ribonucleotide reductase. Prog. Biophys. Mol. Biol..

[6] Wink D.A., Hines H.B., Cheng R.Y., Switzer C.H., Flores-Santana W., Vitek M.P.,  Ridnour L.A., Colton C.A. (2011). Nitric oxide and redox mechanisms in the immune response. J. Leukoc. Biol..

[7] Pierre J.L., Fontecave M. (1999). Iron and activated oxygen species in biology: The basic chemistry. Biometals.

[8] Taketani S. (2005). Aquisition, mobilization and utilization of cellular iron and heme: Endless findings and growing evidence of tight regulation. Tohoku J. Exp. Med..

[9] Micronutrient Deficiencies. http://www.who.int/nutrition/topics/ida/en/.

[10] Theil E.C. (2011). Iron homeostasis and nutritional iron deficiency. J. Nutr..

[11] Shayeghi M., Latunde-Dada G.O., Oakhill J.S., Laftah A.H., Takeuchi K., Halliday N., Khan Y., Warley A., McCann F.E., Hider R.C. (2005). Identification of an intestinal heme transporter. Cell.

[12] Rajagopal A., Rao A.U., Amigo J., Tian M., Upadhyay S.K., Hall C., Uhm S.,  Mathew M.K., Fleming M.D., Paw B.H. (2008). Haem homeostasis is regulated by the conserved and concerted functions of HRG-1 proteins. Nature.

[13] Latunde-Dada G.O., Takeuchi K., Simpson R.J., McKie A.T. (2006). Haem carrier protein 1 (HCP1): Expression and functional studies in cultured cells. FEBS Lett..

[14] Gunshin H., Mackenzie B., Berger U.V., Gunshin Y., Romero M.F., Boron W.F., Nussberger S., Gollan J.L., Hediger M.A. (1997). Cloning and characterization of a mammalian proton-coupled metal-ion transporter. Nature.

[15] Gunshin H., Fujiwara Y., Custodio A.O., Direnzo C., Robine S., Andrews N.C. (2005). Slc11a2 is required for intestinal iron absorption and erythropoiesis but dispensable in placenta and liver. J. Clin. Investig..

[16] McKie A.T., Barrow D., Latunde-Dada G.O., Rolfs A., Sager G., Mudaly E., Mudaly M., Richardson C., Barlow D., Bomford A. (2001). An iron-regulated ferric reductase associated with the absorption of dietary iron. Science.

[17] McKie A.T. (2008). The role of Dcytb in iron metabolism: An update. Biochem. Soc. Trans..

[18] Wyman S., Simpson R.J., McKie A.T., Sharp P.A. (2008). Dcytb (Cybrd1) functions as both a ferric and a cupric reductase *in vitro*. FEBS Lett..

[19] Scheers N. (2013). Regulatory effects of Cu, Zn, and Ca on Fe absorption: The intricate play between nutrient transporters. Nutrients.

[20] Gunshin H., Starr C.N., Direnzo C., Fleming M.D., Jin J., Greer E.L., Sellers V.M., Galica S.M., Andrews N.C. (2005). Cybrd1 (duodenal cytochrome b) is not necessary for dietary iron absorption in mice. Blood.

[21] Chasteen N.D., Harrison P.M. (1999). Mineralization in ferritin: An efficient means of iron storage. J. Struct. Biol..

[22] Miller L.L., Miller S.C., Torti S.V., Tsuji Y., Torti F.M. (1991). Iron-independent induction of ferritin H chain by tumor necrosis factor. Proc. Natl. Acad. Sci. USA.

[23] Leggett B.A., Fletcher L.M., Ramm G.A., Powell L.W., Halliday J.W. (1993). Differential regulation of ferritin H and L subunit mRNA during inflammation and long-term iron overload. J. Gastroenterol. Hepatol..

[24] Shaw G.C., Cope J.J., Li L., Corson K., Hersey C., Ackermann G.E., Gwynn B., Lambert A.J., Wingert R.A., Traver D. (2006). Mitoferrin is essential for erythroid iron assimilation. Nature.

[25] Li F.Y., Nikali K., Gregan J., Leibiger I., Leibiger B., Schweyen R., Larsson C., Suomalainen A. (2001). Characterization of a novel human putative mitochondrial transporter homologous to the yeast mitochondrial RNA splicing proteins 3 and 4. FEBS Lett..

[26] Bencze K.Z., Kondapalli K.C., Cook J.D., McMahon S., Millan-Pacheco C., Pastor N., Stemmler T.L. (2006). The structure and function of frataxin. Crit. Rev. Biochem. Mol. Biol..

[27] Levi S., Arosio P. (2004). Mitochondrial ferritin. Int. J. Biochem. Cell Biol..

[28] Donovan A., Lima C.A., Pinkus J.L., Pinkus G.S., Zon L.I., Robine S., Andrews N.C. (2005). The iron exporter ferroportin/Slc40a1 is essential for iron homeostasis. Cell Metab..

[29] Jeong S.Y., David S. (2003). Glycosylphosphatidylinositol-anchored ceruloplasmin is required for iron efflux from cells in the central nervous system. J. Biol. Chem..

[30] De Domenico I., Ward D.M., di Patti M.C., Jeong S.Y., David S., Musci G., Kaplan J. (2007). Ferroxidase activity is required for the stability of cell surface ferroportin in cells expressing GPI-ceruloplasmin. EMBO J..

[31] Kono S., Yoshida K., Tomosugi N., Terada T., Hamaya Y., Kanaoka S., Miyajima H. (2010). Biological effects of mutant ceruloplasmin on hepcidin-mediated internalization of ferroportin. Biochim. Biophys. Acta.

[32] Han O., Kim E.Y. (2007). Colocalization of ferroportin-1 with hephaestin on the basolateral membrane of human intestinal absorptive cells. J. Cell. Biochem..

[33] Yeh K.Y., Yeh M., Mims L., Glass J. (2009). Iron feeding induces ferroportin 1 and hephaestin migration and interaction in rat duodenal epithelium. Am. J. Physiol. Gastrointest. Liver Physiol..

[34] Aisen P., Leibman A., Zweier J. (1978). Stoichiometric and site characteristics of the binding of iron to human transferrin. J. Biol. Chem..

[35] Ha-Duong N.T., Eid C., Hemadi M., El Hage Chahine J.M. (2010). *In vitro* interaction between ceruloplasmin and human serum transferrin. Biochemistry.

[36] Zakharova E.T., Shavlovski M.M., Bass M.G., Gridasova A.A., Pulina M.O., de Filippis V., Beltramini M., di Muro P., Salvato B., Fontana A. (2000). Interaction of lactoferrin with ceruloplasmin. Arch. Biochem. Biophys..

[37] Hudson D.M., Krisinger M.J., Griffiths T.A., MacGillivray R.T. (2008). Neither human hephaestin nor ceruloplasmin forms a stable complex with transferrin. J. Cell. Biochem..

[38] Dautry-Varsat A., Ciechanover A., Lodish H.F. (1983). pH and the recycling of transferrin during receptor-mediated endocytosis. Proc. Natl. Acad. Sci. USA.

[39] Kawabata H., Germain R.S., Vuong P.T., Nakamaki T., Said J.W., Koeffler H.P. (2000). Transferrin receptor 2-alpha supports cell growth both in iron-chelated cultured cells and *in vivo*. J. Biol. Chem..

[40] Trinder D., Baker E. (2003). Transferrin receptor 2: A new molecule in iron metabolism. Int. J. Biochem. Cell Biol..

[41] Ohgami R.S., Campagna D.R., Greer E.L., Antiochos B., McDonald A., Chen J., Sharp J.J., Fujiwara Y., Barker J.E., Fleming M.D. (2005). Identification of a ferrireductase required for efficient transferrin-dependent iron uptake in erythroid cells. Nat. Genet..

[42] Knutson M.D. (2007). Steap proteins: Implications for iron and copper metabolism. Nutr. Rev..

[43] Liuzzi J.P., Aydemir F., Nam H., Knutson M.D., Cousins R.J. (2006). Zip14 (Slc39a14) mediates non-transferrin-bound iron uptake into cells. Proc. Natl. Acad. Sci. USA.

[44] Christensen E.I., Birn H. (2002). Megalin and cubilin: Multifunctional endocytic receptors. Nat. Rev. Mol. Cell Biol..

[45] Hvidberg V., Jacobsen C., Strong R.K., Cowland J.B., Moestrup S.K., Borregaard N. (2005). The endocytic receptor megalin binds the iron transporting neutrophil-gelatinase-associated lipocalin with high affinity and mediates its cellular uptake. FEBS Lett..

[46] Smith K.D. (2007). Iron metabolism at the host pathogen interface: Lipocalin 2 and the pathogen-associated iroA gene cluster. Int. J. Biochem. Cell Biol..

[47] Devireddy L.R., Gazin C., Zhu X., Green M.R. (2005). A cell-surface receptor for lipocalin 24p3 selectively mediates apoptosis and iron uptake. Cell.

[48] Knutson M., Wessling-Resnick M. (2003). Iron metabolism in the reticuloendothelial system. Crit. Rev. Biochem. Mol. Biol..

[49] Nielsen M.J., Moller H.J., Moestrup S.K. (2010). Hemoglobin and heme scavenger receptors. Antioxid. Redox. Signal..

[50] Troadec M.B., Ward D.M., Kaplan J. (2009). A Tf-independent iron transport system required for organogenesis. Dev. Cell.

[51] Chen T.T., Li L., Chung D.H., Allen C.D., Torti S.V., Torti F.M., Cyster J.G., Chen C.Y., Brodsky F.M., Niemi E.C. (2005). TIM-2 is expressed on B cells and in liver and kidney and is a receptor for H-ferritin endocytosis. J. Exp. Med..

[52] Li L., Fang C.J., Ryan J.C., Niemi E.C., Lebron J.A., Bjorkman P.J., Arase H., Torti F.M., Torti S.V., Nakamura M.C. (2010). Binding and uptake of H-ferritin are mediated by human transferrin receptor-1. Proc. Natl. Acad. Sci. USA.

[53] Mukhopadhyay C.K., Attieh Z.K., Fox P.L. (1998). Role of ceruloplasmin in cellular iron uptake. Science.

[54] Hentze M.W., Muckenthaler M.U., Andrews N.C. (2004). Balancing acts: Molecular control of mammalian iron metabolism. Cell.

[55] Eisenstein R.S., Ross K.L. (2003). Novel roles for iron regulatory proteins in the adaptive response to iron deficiency. J. Nutr..

[56] McKie A.T., Marciani P., Rolfs A., Brennan K., Wehr K., Barrow D., Miret S., Bomford A., Peters T.J., Farzaneh F. (2000). A novel duodenal iron-regulated transporter, IREG1, implicated in the basolateral transfer of iron to the circulation. Mol. Cell.

[57] Zhang D.L., Hughes R.M., Ollivierre-Wilson H., Ghosh M.C., Rouault T.A. (2009). A ferroportin transcript that lacks an iron-responsive element enables duodenal and erythroid precursor cells to evade translational repression. Cell Metab..

[58] Lee P.L., Gelbart T., West C., Halloran C., Beutler E. (1998). The human Nramp2 gene: Characterization of the gene structure, alternative splicing, promoter region and polymorphisms. Blood Cells Mol. Dis..

[59] Meyron-Holtz E.G., Ghosh M.C., Iwai K., LaVaute T., Brazzolotto X., Berger U.V., Land W., Ollivierre-Wilson H., Grinberg A., Love P. (2004). Genetic ablations of iron regulatory proteins 1 and 2 reveal why iron regulatory protein 2 dominates iron homeostasis. EMBO J..

[60] Smith S.R., Ghosh M.C., Ollivierre-Wilson H., Tong W.H., Rouault T.A. (2006). Complete loss of iron regulatory proteins 1 and 2 prevents viability of murine zygotes beyond the blastocyst stage of embryonic development. Blood Cells Mol. Dis..

[61] Fleming R.E., Sly W.S. (2001). Hepcidin: A putative iron-regulatory hormone relevant to hereditary hemochromatosis and the anemia of chronic disease. Proc. Natl. Acad. Sci. USA.

[62] Nemeth E., Tuttle M.S., Powelson J., Vaughn M.B., Donovan A., Ward D.M., Ganz T., Kaplan J. (2004). Hepcidin regulates cellular iron efflux by binding to ferroportin and inducing its internalization. Science.

[63] Bardou-Jacquet E., Island M.L., Jouanolle A.M., Detivaud L., Fatih N., Ropert M., Brissot E., Mosser A., Maisonneuve H., Brissot P. (2011). A novel N491S mutation in the human SLC11A2 gene impairs protein trafficking and in association with the G212V mutation leads to microcytic anemia and liver iron overload. Blood Cells Mol. Dis..

[64] Beaumont C., Delaunay J., Hetet G., Grandchamp B., de Montalembert M., Tchernia G. (2006). Two new human DMT1 gene mutations in a patient with microcytic anemia, low ferritinemia, and liver iron overload. Blood.

[65] Blanco E., Kannengiesser C., Grandchamp B., Tasso M., Beaumont C. (2009). Not all DMT1 mutations lead to iron overload. Blood Cells Mol. Dis..

[66] Kato J., Fujikawa K., Kanda M., Fukuda N., Sasaki K., Takayama T., Kobune M., Takada K., Takimoto R., Hamada H. (2001). A mutation, in the iron-responsive element of H ferritin mRNA, causing autosomal dominant iron overload. Am. J. Hum. Genet..

[67] Curtis A.R., Fey C., Morris C.M., Bindoff L.A., Ince P.G., Chinnery P.F., Coulthard A., Jackson M.J., Jackson A.P., McHale D.P. (2001). Mutation in the gene encoding ferritin light polypeptide causes dominant adult-onset basal ganglia disease. Nat. Genet..

[68] Levi S., Cozzi A., Arosio P. (2005). Neuroferritinopathy: A neurodegenerative disorder associated with l-ferritin mutation. Best Pract. Res. Clin. Haematol..

[69] Cazzola M., Bergamaschi G., Tonon L., Arbustini E., Grasso M., Vercesi E., Barosi G., Bianchi P.E., Cairo G., Arosio P. (1997). Hereditary hyperferritinemia-cataract syndrome: Relationship between phenotypes and specific mutations in the iron-responsive element of ferritin light-chain mRNA. Blood.

[70] Koeppen A.H. (2011). Friedreich’s ataxia: Pathology, pathogenesis, and molecular genetics. J. Neurol. Sci..

[71] De Domenico I., Ward D.M., Nemeth E., Vaughn M.B., Musci G., Ganz T., Kaplan J. (2005). The molecular basis of ferroportin-linked hemochromatosis. Proc. Natl. Acad. Sci. USA.

[72] Xu X., Pin S., Gathinji M., Fuchs R., Harris Z.L. (2004). Aceruloplasminemia: An inherited neurodegenerative disease with impairment of iron homeostasis. Ann. N. Y. Acad. Sci..

[73] Hamill R.L., Woods J.C., Cook B.A. (1991). Congenital atransferrinemia. A case report and review of the literature. Am. J. Clin. Pathol..

[74] Shamsian B.S., Rezaei N., Arzanian M.T., Alavi S., Khojasteh O., Eghbali A. (2009). Severe hypochromic microcytic anemia in a patient with congenital atransferrinemia. Pediatr. Hematol. Oncol..

[75] Chen J., Enns C.A. (2011). Hereditary hemochromatosis and transferrin receptor 2. Biochim. Biophys. Acta.

[76] Rochette J., le Gac G., Lassoued K., Ferec C., Robson K.J. (2010). Factors influencing disease phenotype and penetrance in HFE haemochromatosis. Hum. Genet..

[77] Goldberg Y.P., Pagon R.A., Bird T.D., Dolan C.R., Stephens K., Adam M.P. (2011). Juvenile Hereditary Hemochromatosis. GeneReviews™ [Internet].

[78] Sakurai T., Kataoka K. (2007). Basic and applied features of multicopper oxidases, CueO, bilirubin oxidase, and laccase. Chem. Rec..

[79] Kosman D.J. (2010). Multicopper oxidases: A workshop on copper coordination chemistry, electron transfer, and metallophysiology. J. Biol. Inorg. Chem..

[80] Nakamura K., Kawabata T., Yura K., Go N. (2003). Novel types of two-domain multi-copper oxidases: Possible missing links in the evolution. FEBS Lett..

[81] Skalova T., Dohnalek J., Ostergaard L.H., Ostergaard P.R., Kolenko P., Duskova J., Stepankova A., Hasek J. (2009). The structure of the small laccase from Streptomyces coelicolor reveals a link between laccases and nitrite reductases. J. Mol. Biol..

[82] Nakamura K., Go N. (2005). Function and molecular evolution of multicopper blue proteins. Cell. Mol. Life Sci..

[83] Giardina P., Faraco V., Pezzella C., Piscitelli A., Vanhulle S., Sannia G. (2009). Laccases: A never-ending story. Cell. Mol. Life Sci..

[84] Hirose J., Sakurai T., Imamura K., Watanabe H., Iwamoto H., Hiromi K., Itoh H., Shin T., Murao S. (1994). Characterization of ascorbate oxidase from *Acremonium* sp. HI-25. J. Biochem..

[85] Terzulli A., Kosman D.J. (2010). Analysis of the high-affinity iron uptake system at the *Chlamydomonas reinhardtii* plasma membrane. Eukaryot. Cell.

[86] Roberts S.A., Weichsel A., Grass G., Thakali K., Hazzard J.T., Tollin G., Rensing C., Montfort W.R. (2002). Crystal structure and electron transfer kinetics of CueO, a multicopper oxidase required for copper homeostasis in *Escherichia coli*. Proc. Natl. Acad. Sci. USA.

[87] Dick G.J., Torpey J.W., Beveridge T.J., Tebo B.M. (2008). Direct identification of a bacterial manganese(II) oxidase, the multicopper oxidase MnxG, from spores of several different marine Bacillus species. Appl. Environ. Microbiol..

[88] Palmer A.E., Szilagyi R.K., Cherry J.R., Jones A., Xu F., Solomon E.I. (2003). Spectroscopic characterization of the Leu513His variant of fungal laccase: Effect of increased axial ligand interaction on the geometric and electronic structure of the type 1 Cu site. Inorg. Chem..

[89] Xu F., Palmer A.E., Yaver D.S., Berka R.M., Gambetta G.A., Brown S.H., Solomon E.I. (1999). Targeted mutations in a *Trametes villosa* laccase. Axial perturbations of the T1 copper. J. Biol. Chem..

[90] Solomon E.I., Szilagyi R.K., DeBeer George S., Basumallick L. (2004). Electronic structures of metal sites in proteins and models: Contributions to function in blue copper proteins. Chem. Rev..

[91] Quintanar L., Stoj C., Taylor A.B., Hart P.J., Kosman D.J., Solomon E.I. (2007). Shall we dance? How a multicopper oxidase chooses its electron transfer partner. Acc. Chem. Res..

[92] Solomon E.I. (2006). Spectroscopic methods in bioinorganic chemistry: Blue to green to red copper sites. Inorg. Chem..

[93] Sakurai T., Kataoka K. (2007). Structure and function of type I copper in multicopper oxidases. Cell. Mol. Life Sci..

[94] Quintanar L., Stoj C., Wang T.P., Kosman D.J., Solomon E.I. (2005). Role of aspartate 94 in the decay of the peroxide intermediate in the multicopper oxidase Fet3p. Biochemistry.

[95] Yoon J., Solomon E.I. (2007). Electronic structure of the peroxy intermediate and its correlation to the native intermediate in the multicopper oxidases: Insights into the reductive cleavage of the O–O bond. J. Am. Chem. Soc..

[96] Yoon J., Liboiron B.D., Sarangi R., Hodgson K.O., Hedman B., Solomon E.I. (2007). The two oxidized forms of the trinuclear Cu cluster in the multicopper oxidases and mechanism for the decay of the native intermediate. Proc. Natl. Acad. Sci. USA.

[97] Solomon E.I., Augustine A.J., Yoon J. (2008). O_2_ reduction to H_2_O by the multicopper oxidases. Dalton Trans..

[98] Holmberg C.G., Laurell C.B. (1948). Investigations in serum copper. II. Isolation of copper containing protein, and a description of some of its properties. Acta Chem. Scand..

[99] Osaki S., Johnson D.A., Frieden E. (1966). The possible significance of the ferrous oxidase activity of ceruloplasmin in normal human serum. J. Biol. Chem..

[100] Healy J., Tipton K. (2007). Ceruloplasmin and what it might do. J. Neural Transm..

[101] Patel B.N., David S. (1997). A novel glycosylphosphatidylinositol-anchored form of ceruloplasmin is expressed by mammalian astrocytes. J. Biol. Chem..

[102] Hahn P., Qian Y., Dentchev T., Chen L., Beard J., Harris Z.L., Dunaief J.L. (2004). Disruption of ceruloplasmin and hephaestin in mice causes retinal iron overload and retinal degeneration with features of age-related macular degeneration. Proc. Natl. Acad. Sci. USA.

[103] Fortna R.R., Watson H.A., Nyquist S.E. (1999). Glycosyl phosphatidylinositol-anchored ceruloplasmin is expressed by rat Sertoli cells and is concentrated in detergent-insoluble membrane fractions. Biol. Reprod..

[104] Vachette P., Dainese E., Vasyliev V.B., di Muro P., Beltramini M., Svergun D.I., de Filippis V., Salvato B. (2002). A key structural role for active site type 3 copper ions in human ceruloplasmin. J. Biol. Chem..

[105] Lindley P.F., Card G., Zaitseva I., Zaitsev V., Reinhammar B., Selin-Lindgren E., Yoshida K. (1997). An X-ray structural study of human ceruloplasmin in relation to ferroxidase activity. J. Biol. Inorg. Chem..

[106] Quintanar L., Gebhard M., Wang T.P., Kosman D.J., Solomon E.I. (2004). Ferrous binding to the multicopper oxidases *Saccharomyces cerevisiae* Fet3p and human ceruloplasmin: Contributions to ferroxidase activity. J. Am. Chem. Soc..

[107] Machonkin T.E., Zhang H.H., Hedman B., Hodgson K.O., Solomon E.I. (1998). Spectroscopic and magnetic studies of human ceruloplasmin: Identification of a redox-inactive reduced type 1 copper site. Biochemistry.

[108] Brown M.A., Stenberg L.M., Mauk A.G. (2002). Identification of catalytically important amino acids in human ceruloplasmin by site-directed mutagenesis. FEBS Lett..

[109] Inoue K., Akaike T., Miyamoto Y., Okamoto T., Sawa T., Otagiri M., Suzuki S., Yoshimura T., Maeda H. (1999). Nitrosothiol formation catalyzed by ceruloplasmin. Implication for cytoprotective mechanism *in vivo*. J. Biol. Chem..

[110] Cha M.K., Kim I.H. (1999). Ceruloplasmin has a distinct active site for the catalyzing glutathione-dependent reduction of alkyl hydroperoxide. Biochemistry.

[111] Stoj C., Kosman D.J. (2003). Cuprous oxidase activity of yeast Fet3p and human ceruloplasmin: Implication for function. FEBS Lett..

[112] Mukhopadhyay C.K., Mazumder B., Lindley P.F., Fox P.L. (1997). Identification of the prooxidant site of human ceruloplasmin: A model for oxidative damage by copper bound to protein surfaces. Proc. Natl. Acad. Sci. USA.

[113] Young S.N., Curzon G. (1972). A method for obtaining linear reciprocal plots with caeruloplasmin and its application in a study of the kinetic parameters of caeruloplasmin substrates. Biochem. J..

[114] McDermott J.A., Huber C.T., Osaki S., Frieden E. (1968). Role of iron in the oxidase activity of ceruloplasmin. Biochim. Biophys. Acta..

[115] Zaitsev V.N., Zaitseva I., Papiz M., Lindley P.F. (1999). An X-ray crystallographic study of the binding sites of the azide inhibitor and organic substrates to ceruloplasmin, a multi-copper oxidase in the plasma. J. Biol. Inorg. Chem..

[116] Harris Z.L., Takahashi Y., Miyajima H., Serizawa M., MacGillivray R.T., Gitlin J.D. (1995). Aceruloplasminemia: Molecular characterization of this disorder of iron metabolism. Proc. Natl. Acad. Sci. USA.

[117] Yazaki M., Yoshida K., Nakamura A., Furihata K., Yonekawa M., Okabe T., Yamashita N., Ohta M., Ikeda S. (1998). A novel splicing mutation in the ceruloplasmin gene responsible for hereditary ceruloplasmin deficiency with hemosiderosis. J. Neurol. Sci..

[118] Kono S., Suzuki H., Oda T., Shirakawa K., Takahashi Y., Kitagawa M., Miyajima H. (2007). Cys-881 is essential for the trafficking and secretion of truncated mutant ceruloplasmin in aceruloplasminemia. J. Hepatol..

[119] Hellman N.E., Kono S., Miyajima H., Gitlin J.D. (2002). Biochemical analysis of a missense mutation in aceruloplasminemia. J. Biol. Chem..

[120] Kono S., Suzuki H., Takahashi K., Takahashi Y., Shirakawa K., Murakawa Y., Yamaguchi S., Miyajima H. (2006). Hepatic iron overload associated with a decreased serum ceruloplasmin level in a novel clinical type of aceruloplasminemia. Gastroenterology.

[121] Patel B.N., Dunn R.J., Jeong S.Y., Zhu Q., Julien J.P., David S. (2002). Ceruloplasmin regulates iron levels in the CNS and prevents free radical injury. J. Neurosci..

[122] Vulpe C.D., Kuo Y.M., Murphy T.L., Cowley L., Askwith C., Libina N., Gitschier J., Anderson G.J. (1999). Hephaestin, a ceruloplasmin homologue implicated in intestinal iron transport, is defective in the sla mouse. Nat. Genet..

[123] Edwards J.A., Bannerman R.M. (1970). Hereditary defect of intestinal iron transport in mice with sex-linked anemia. J. Clin. Investig..

[124] Syed B.A., Beaumont N.J., Patel A., Naylor C.E., Bayele H.K., Joannou C.L., Rowe P.S., Evans R.W., Srai S.K. (2002). Analysis of the human hephaestin gene and protein: Comparative modelling of the *N*-terminus ecto-domain based upon ceruloplasmin. Protein Eng..

[125] Frazer D.M., Vulpe C.D., McKie A.T., Wilkins S.J., Trinder D., Cleghorn G.J., Anderson G.J. (2001). Cloning and gastrointestinal expression of rat hephaestin: Relationship to other iron transport proteins. Am. J. Physiol. Gastrointest. Liver Physiol..

[126] Hudson D.M., Curtis S.B., Smith V.C., Griffiths T.A., Wong A.Y., Scudamore C.H., Buchan A.M., MacGillivray R.T. (2010). Human hephaestin expression is not limited to enterocytes of the gastrointestinal tract but is also found in the antrum, the enteric nervous system, and pancreatic β-cells. Am. J. Physiol. Gastrointest. Liver Physiol..

[127] Qian Z.M., Chang Y.Z., Leung G., Du J.R., Zhu L., Wang Q., Niu L., Xu Y.J., Yang L., Ho K.P. (2007). Expression of ferroportin1, hephaestin and ceruloplasmin in rat heart. Biochim. Biophys. Acta.

[128] Qian Z.M., Chang Y.Z., Zhu L., Yang L., Du J.R., Ho K.P., Wang Q., Li L.Z., Wang C.Y., Ge X. (2007). Development and iron-dependent expression of hephaestin in different brain regions of rats. J. Cell. Biochem..

[129] Kingston P.J., Bannerman C.E., Bannerman R.M. (1978). Iron deficiency anaemia in newborn sla mice: A genetic defect of placental iron transport. Br. J. Haematol..

[130] Vashchenko G., Bleackley M.R., Griffiths T.A., MacGillivray R.T. (2011). Oxidation of organic and biogenic amines by recombinant human hephaestin expressed in *Pichia pastoris*. Arch. Biochem. Biophys..

[131] Vashchenko G., Macgillivray R.T. (2012). Functional role of the putative iron ligands in the ferroxidase activity of recombinant human hephaestin. J. Biol. Inorg. Chem..

[132] Chen H., Attieh Z.K., Syed B.A., Kuo Y.M., Stevens V., Fuqua B.K., Andersen H.S., Naylor C.E., Evans R.W., Gambling L. (2010). Identification of zyklopen, a new member of the vertebrate multicopper ferroxidase family, and characterization in rodents and human cells. J. Nutr..

[133] Danzeisen R., Ponnambalam S., Lea R.G., Page K., Gambling L., McArdle H.J. (2000). The effect of ceruloplasmin on iron release from placental (BeWo) cells; evidence for an endogenous Cu oxidase. Placenta.

[134] Danzeisen R., Fosset C., Chariana Z., Page K., David S., McArdle H.J. (2002). Placental ceruloplasmin homolog is regulated by iron and copper and is implicated in iron metabolism. Am. J. Physiol. Cell Physiol..

[135] Cui R., Duan X.L., Anderson G.J., Qiao Y.T., Yu P., Qian Z.M., Yoshida K., Takeda S., Guo P., Yang Z.L. (2009). Age-dependent expression of hephaestin in the brain of ceruloplasmin-deficient mice. J. Trace Elem. Med. Biol..

[136] Harris Z.L., Durley A.P., Man T.K., Gitlin J.D. (1999). Targeted gene disruption reveals an essential role for ceruloplasmin in cellular iron efflux. Proc. Natl. Acad. Sci. USA.

[137] Bannerman R.M., Cooper R.G. (1966). Sex-linked anemia: A hypochromic anemia of mice. Science.

[138] Li Y.Q., Bai B., Cao X.X., Yan H., Zhuang G.H. (2012). Ferroportin 1 and hephaestin expression in BeWo cell line with different iron treatment. Cell Biochem. Funct..

[139] Cherukuri S., Potla R., Sarkar J., Nurko S., Harris Z.L., Fox P.L. (2005). Unexpected role of ceruloplasmin in intestinal iron absorption. Cell Metab..

[140] Mukhopadhyay C.K., Mazumder B., Fox P.L. (2000). Role of hypoxia-inducible factor-1 in transcriptional activation of ceruloplasmin by iron deficiency. J. Biol. Chem..

[141] Tapryal N., Mukhopadhyay C., Das D., Fox P.L., Mukhopadhyay C.K. (2009). Reactive oxygen species regulate ceruloplasmin by a novel mRNA decay mechanism involving its 3′-untranslated region: Implications in neurodegenerative diseases. J. Biol. Chem..

[142] Sampath P., Mazumder B., Seshadri V., Fox P.L.  (2003). Transcript-selective translational silencing by gamma interferon is directed by a novel structural element in the ceruloplasmin mRNA 3′ untranslated region. Mol. Cell. Biol..

[143] Persichini T., Maio N., di Patti M.C., Rizzo G., Toscano S., Colasanti M., Musci G. (2010). Interleukin-1beta induces ceruloplasmin and ferroportin-1 gene expression via MAP kinases and C/EBPbeta, AP-1, and NF-kappaB activation. Neurosci. Lett..

[144] Chen H., Su T., Attieh Z.K., Fox T.C., McKie A.T., Anderson G.J., Vulpe C.D. (2003). Systemic regulation of Hephaestin and Ireg1 revealed in studies of genetic and nutritional iron deficiency. Blood.

[145] Lee S.M., Attieh Z.K., Son H.S., Chen H., Bacouri-Haidar M., Vulpe C.D. (2012). Iron repletion relocalizes hephaestin to a proximal basolateral compartment in polarized MDCK and Caco2 cells. Biochem. Biophys. Res. Commun..

[146] Hinoi T., Gesina G., Akyol A., Kuick R., Hanash S., Giordano T.J., Gruber S.B., Fearon E.R. (2005). CDX2-regulated expression of iron transport protein hephaestin in intestinal and colonic epithelium. Gastroenterology.

[147] Nittis T., Gitlin J.D. (2004). Role of copper in the proteosome-mediated degradation of the multicopper oxidase hephaestin. J. Biol. Chem..

[148] Chen H., Huang G., Su T., Gao H., Attieh Z.K., McKie A.T., Anderson G.J., Vulpe C.D. (2006). Decreased hephaestin activity in the intestine of copper-deficient mice causes systemic iron deficiency. J. Nutr..

[149] Gitlin J.D., Schroeder J.J., Lee-Ambrose L.M., Cousins R.J. (1992). Mechanisms of caeruloplasmin biosynthesis in normal and copper-deficient rats. Biochem. J..

